# Global analysis of protein aggregation in yeast during physiological conditions and arsenite stress

**DOI:** 10.1242/bio.20148938

**Published:** 2014-09-12

**Authors:** Sebastian Ibstedt, Theodora C. Sideri, Chris M. Grant, Markus J. Tamás

**Affiliations:** 1 Department of Chemistry and Molecular Biology, University of Gothenburg, S-405 30 Gothenburg, Sweden; 2 Faculty of Life Sciences, University of Manchester, Manchester M13 9PT, UK; 3 Current address: Department of Genetics, Evolution and Environment and UCL Cancer Institute, University College London, WC1E 6BT, London, UK.

**Keywords:** protein aggregation, protein folding, translation, arsenite, chaperone

## Abstract

Protein aggregation is a widespread phenomenon in cells and associated with pathological conditions. Yet, little is known about the rules that govern protein aggregation in living cells. In this study, we biochemically isolated aggregation-prone proteins and used computational analyses to identify characteristics that are linked to physiological and arsenite-induced aggregation in living yeast cells. High protein abundance, extensive physical interactions, and certain structural properties are positively correlated with an increased aggregation propensity. The aggregated proteins have high translation rates and are substrates of ribosome-associated Hsp70 chaperones, indicating that they are susceptible for aggregation primarily during translation/folding. The aggregation-prone proteins are enriched for multiple chaperone interactions, thus high protein abundance is probably counterbalanced by molecular chaperones to allow soluble expression *in vivo*. Our data support the notion that arsenite interferes with chaperone activity and indicate that arsenite-aggregated proteins might engage in extensive aberrant protein–protein interactions. Expression of aggregation-prone proteins is down-regulated during arsenite stress, possibly to prevent their toxic accumulation. Several aggregation-prone yeast proteins have human homologues that are implicated in misfolding diseases, suggesting that similar mechanisms may apply in disease- and non-disease settings.

## INTRODUCTION

Proteins participate in virtually every biological process. To function, most proteins fold into a strictly defined three-dimensional structure, their native conformation. Proteins in a non-native conformation may aggregate and/or engage in aberrant interactions with other cellular components. Misfolded proteins are cytotoxic, and numerous neurodegenerative and age-related disorders are associated with protein misfolding and aggregation. Evolutionary conserved protein quality-control (PQC) systems protect cells against the harmful accumulation of protein aggregates. These PQC systems encompass molecular chaperones that assist folding of polypeptides into their functional conformation and degradation pathways that clear the cells from misfolded and aggregated proteins (Hartl et al., 2011; Stefani and Dobson, 2003; Tyedmers et al., 2010).

The inclination of a given protein to aggregate is correlated with solvent-exposed stretches of high hydrophobicity, high β-sheet propensity, and a low net charge (Hartl et al., 2011; Stefani and Dobson, 2003). Moreover, structurally flexible proteins and proteins with intrinsically disordered regions may be more prone to aberrant interactions and aggregation (Breydo and Uversky, 2011). Aggregation-prone segments tend to be buried in the native (folded) protein. However, conditions that promote protein unfolding may lead to exposure of such segments and facilitate aggregation. Such conditions include mutations that affect the PQC systems, misprocessing phenomena such as mistranslation or defective assembly of protein complexes, changes in the intracellular environment or chemical modifications, and progressive decline in efficiency of the PQC systems during ageing (Hartl et al., 2011; Stefani and Dobson, 2003; Tyedmers et al., 2010).

Much of our knowledge on protein folding and aggregation comes from *in vitro* studies using model peptides, and misfolding-prone or disease-associated (model) proteins (Alies et al., 2013; Breydo and Uversky, 2011; Hartl et al., 2011; Vendruscolo, 2012). In addition, computational approaches are commonly used to predict the intrinsic aggregation-propensities of proteins (Conchillo-Solé et al., 2007; Fernandez-Escamilla et al., 2004; Tartaglia and Vendruscolo, 2008). In general, the algorithms used are based on certain physico-chemical characteristics of the amino acid sequence previously shown to contribute to protein aggregation (using *in vitro* measurements). However, the rules that govern protein aggregation in living cells are likely to be more complex than those defined from individual proteins or from *in vitro* studies (Vendruscolo, 2012). Recently, proteome-wide studies on aggregation in living cells have been reported. For example, it was estimated that hundreds of proteins aggregate upon mild heat stress in *Escherichia coli* (Winkler et al., 2010). Likewise, about 200 aggregated proteins were identified in stationary phase yeast (*Saccharomyces cerevisiae*) cells. For a subset of those proteins, the process of aggregation was reversible upon nutrient re-addition (Narayanaswamy et al., 2009). Similarly, numerous proteins turned insoluble with age in *Caenorhabditis elegans* (David et al., 2010) or co-aggregated with amyloid-forming polypeptides in mammalian cells (Olzscha et al., 2011). Widespread protein aggregation also occurs in cells defective in PQC systems (Chapman et al., 2006; Koplin et al., 2010; Rand and Grant, 2006), in response to environmental stress conditions (Jacobson et al., 2012), and in disease processes (Basso et al., 2009; Liao et al., 2004; Wang et al., 2005; Xia et al., 2008).

There is accumulating evidence that certain metals influence the aggregation propensity of disease-associated proteins and affect the progression of certain neurodegenerative diseases via largely unknown mechanisms (Alies et al., 2013; Bourassa and Miller, 2012; Breydo and Uversky, 2011; Caudle et al., 2012; Savelieff et al., 2013). Recent studies showed that various metals and the metalloid arsenite inhibit protein folding *in vitro* (Jacobson et al., 2012; Ramadan et al., 2009; Sharma et al., 2008; Tamás et al., 2014). Moreover, we demonstrated that arsenite interferes with protein folding *in vivo* by acting on unfolded or nascent polypeptides and by directly interfering with chaperone activity (Jacobson et al., 2012). Folding inhibition contributed to arsenite toxicity in two ways; by aggregate formation and by chaperone inhibition. Interestingly, *in vitro* data indicated that arsenite-induced protein aggregates can act as seeds committing other, labile proteins to misfold and aggregate (Jacobson et al., 2012). This mode of action may explain the suggested role of this metalloid in the etiology of certain neurodegenerative and age-related disorders associated with arsenic poisoning. However, much remains to be learned about the molecular events leading to protein aggregation and aggregate toxicity in living cells. In this study, we addressed the following questions: (1) What proteins are at risk for aggregation *in vivo*? (2) What physico-chemical properties and biological functions are associated with protein aggregation? (3) How do aggregates contribute to arsenite toxicity? (4) Do cells regulate aggregation-prone proteins during environmental stress? For this, we biochemically isolated aggregated proteins from *S. cerevisiae* during physiological conditions and arsenite exposure, and used computational analyses to identify characteristics that are linked to protein aggregation. In this way, we provide novel and extended insights into the rules that govern protein aggregation in living cells.

## MATERIALS AND METHODS

### Identification of aggregated proteins

Yeast cells (BY4742 strain background) were grown to exponential phase (A_600_ ∼0.6) in YPD medium without or with arsenite (1.5 mM sodium arsenite, 1 hour) and equivalent cell numbers (10 A_600_ units) were used to isolate aggregated proteins as described previously (Jacobson et al., 2012; Rand and Grant, 2006). Briefly, cells were disrupted in lysis buffer (50 mM potassium phosphate buffer, pH 7, 1 mM EDTA, 5% glycerol, 1 mM phenylmethylsulfonyl fluoride and Complete Mini protease inhibitor cocktail (Roche)), and membrane proteins and aggregated proteins were isolated by centrifugation (15,000 *g*; 20 minutes). Membrane proteins were removed by washing twice with 320 µl lysis buffer and 80 µl of 10% Igepal CA 630 (NP-40) (Sigma–Aldrich), centrifuging at 15,000 *g* for 20 minutes each time, and the final aggregated protein extract was resuspended in 100 µl of lysis buffer. Aggregated proteins were separated on 12% reducing SDS-PAGE gels and stained using colloidal Coomassie blue (Sigma–Aldrich). Proteins were excised, trypsin-digested, and identified using liquid chromatography-mass spectrometry (LC-MS) in the Biomolecular Analysis Facility (Faculty of Life Sciences, University of Manchester). Proteins were identified using the Mascot mass fingerprinting programme (http://www.matrixscience.com) to search the NCBInr and Swissprot databases.

### Statistical methods

Statistical analyses were performed on physiological aggregates (P-set) and on arsenite-induced aggregates (As-set) using a largely unbiased set of 1475 proteins (MS proteome) detected by large-scale proteome analysis by multidimensional LC-MS (Washburn et al., 2001) as background.

### Analyses of physical properties

Analysis of functional enrichment was performed on gene ontology data from the *Saccharomyces* Genome Database (SGD) (Cherry et al., 2012) and *p*-values were calculated with a hypergeometric test using 6607 genomic genes as background. *p*-values were filtered with FDR ≤ 5%. Physical protein properties were obtained from SGD and analysed with Mann–Whitney U-tests.

### Analyses of protein abundance, translation, expression, and half-lives

Protein abundance (Ghaemmaghami et al., 2003), translation rate per protein species (Arava et al., 2003) and protein half-life (Belle et al., 2006) was analysed based on data collected during non-stress conditions. Mann–Whiney U-tests were used to assess the observed differences. Genome-wide expression data was obtained from (Thorsen et al., 2007). Overlap between proteins showing at least a 2-fold change in gene expression and aggregated proteins in the As- and P-sets was evaluated with a hypergeometric test, using the MS proteome as background. The representation factor was calculated as observed overlap/expected overlap.

### Analyses of structural properties

Secondary structures were predicted with the Garnier–Osguthorpe–Robson algorithm (Garnier et al., 1978). For each protein, the relative proportion of amino acid residues partaking in a particular secondary structure (α-helix, β-sheet) was predicted using a sliding 17 residue window. Results were confirmed by an alternative approach based on homologies with known structures (Frishman and Argos, 1995; Frishman and Argos, 1997). Proportions of residues in secondary structures were compared with Mann–Whitney U-tests. Intrinsic disorder was predicted by calculating the fold-index, defined as a function of mean hydrophobicity and mean net charge (Prilusky et al., 2005). The genomic proportion of surface-exposed cysteines was identified by (Marino et al., 2010) and used to compare the As- and P-sets to the MS proteome with Fisher's exact test. Cysteine density was calculated by counting the number of CC, CxC, CxxC or CxxxC in a sliding window across each protein. *p*-values were computed with Fisher's exact test. Differences in amino acid composition were compared with heteroscedastic Student's t-test and adjusted for multiple testing with Holm–Šídák correction.

### Analyses of interactivity

Genetic and physical interaction data were obtained from the BioGRID database (Stark et al., 2006) and subset on group-specific and global interactions for proteins in both aggregate sets. Data for calculation of synthetic sickness was obtained from the Drygin database (Koh et al., 2010) and filtered for maximum 5% false positives. A difference of at least 0.08 between the fitness of the double mutant and the two single mutants, |*f_ab_*−*f_a_*×*f_b_*|>0.08, was considered to display synthetic sickness, a definition which has proven to give reproducible and functionally informative results (Koh et al., 2010). Differences between the data-sets with regard to physical, genetic and synthetic sick interactions were assessed by empirical *p*-values by comparing the median difference between observed groups with 1,000,000 random permutations (without replacement) of the pooled data. Interactions with chaperones were based on data from (Gong et al., 2009) and results were analyzed with Student's t-test. Overrepresentation of co-translational Ssb2p substrates and aggregation in *SSBΔ* cells (Willmund et al., 2013) was analyzed with Fisher's exact test.

### Identification of orthologues

Orthologues between human disease aggregates in Alzheimer's disease (Liao et al., 2004; Wang et al., 2005), familial amyotrophic lateral sclerosis (Basso et al., 2009) or Parkinson's disease (Xia et al., 2008) and yeast were identified with the OMA browser (Schneider et al., 2007). The level of orthology was evaluated by counting the number of orthologous cases between disease-associated aggregates and the yeast aggregates and the genome, respectively, and assessed with Fisher's exact test.

## RESULTS

### Identification of aggregation-prone proteins in *S. cerevisiae*

To identify aggregation-prone proteins, we collected exponentially growing yeast cells that were either untreated (physiological condition) or exposed to sodium arsenite. Aggregated proteins were isolated using a well-established method based on density centrifugation, and then identified using LC-MS (see Materials and Methods). In this way, a total of 257 aggregated proteins were unambiguously identified (supplementary material Table S3). Of these, 114 proteins were found to aggregate both under physiological conditions and during arsenite exposure; these proteins are likely to be generally aggregation-prone and will hereafter be referred to as the physiological-set (P-set). Since the MS method used is qualitative, our data-sets do not contain information whether a greater percentage of a given protein in the P-set would aggregate during arsenite exposure. The remaining 143 proteins were unique to arsenite-exposed cells (hereafter called the As-set). Thus, an expanded set of proteins aggregate in response to arsenite exposure. We previously reported the identity of the proteins in the As-set (Jacobson et al., 2012); however, that report did not include systems-level analysis of the aggregated proteins. In this current study, we performed a comprehensive analysis of aggregation-prone proteins during physiological conditions and arsenite stress.

### Physical, structural and functional characteristics of aggregation-prone proteins

**Fig. 1.**
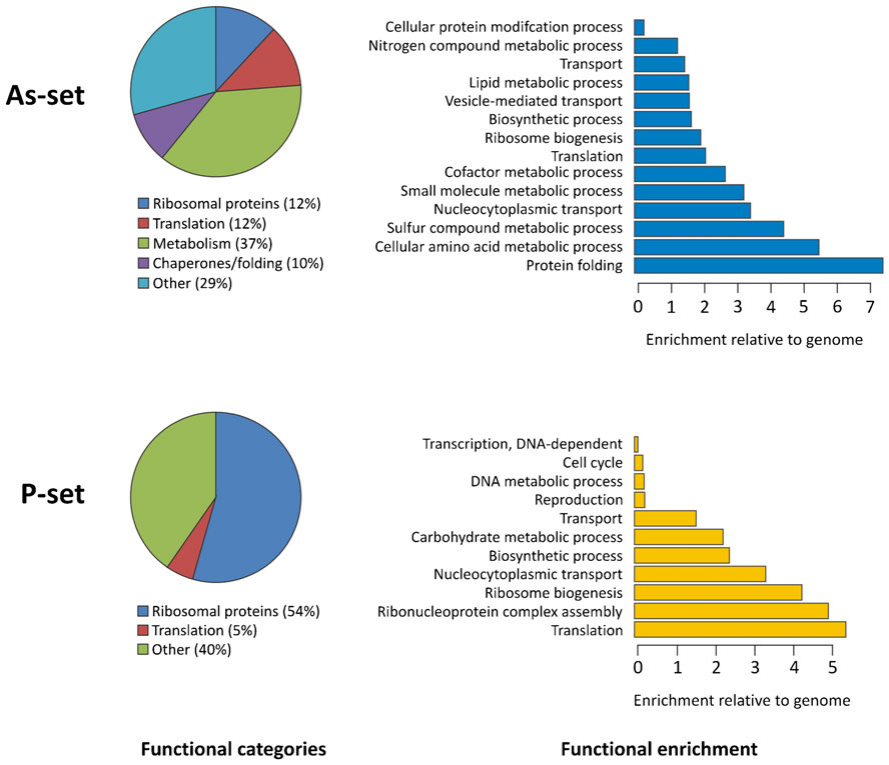
Functional characteristics of aggregation-prone proteins. Biological processes that are significantly enriched in the As- and P-sets compared to the *S. cerevisiae* genome. Circle diagrams indicate the distribution of aggregated proteins into functional categories where “Translation” excludes ribosomal proteins. Bar diagrams indicate the fold-enrichment of functional categories compared to the genome using GO data from SGD. All shown categories are significant with 5% FDR.

Gene ontology (GO) analysis indicated that aggregated proteins are enriched in certain functional categories (Fig. 1). Functions related to ribosome biogenesis/assembly and translation are highly overrepresented in the P-set compared to the *S. cerevisiae* genome. In fact, about 60% of the proteins in the P-set are ribosomal proteins or have a role during translation. The As-set is enriched for functions related to translation, protein folding, and various metabolic processes (Fig. 1).

**Fig. 2.**
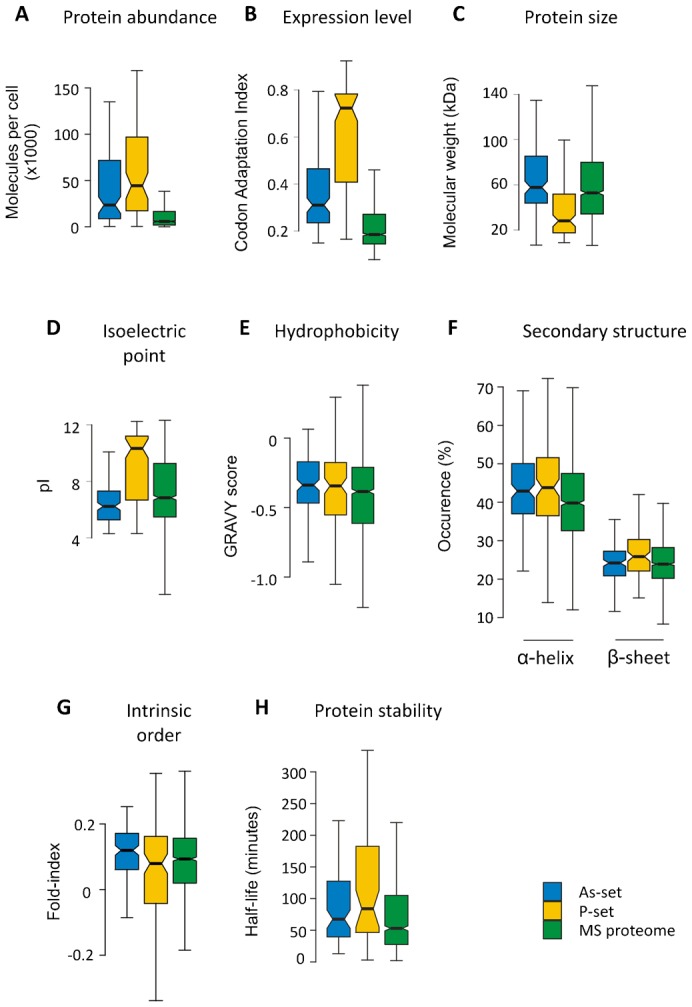
Physico-chemical characteristics of aggregation-prone proteins. (A) Molecules per cell. The abundance of proteins in each set during non-stress conditions is plotted. Proteins in the P-set are significantly more abundant than proteins in the As-set (*p*  =  0.017, *U*  =  3907.5), whilst proteins in the MS proteome are less abundant than those in the As-set (*p* < 10^−15^, *U*  =  100021) and P-set (*p* < 10^−15^, *U*  =  69411). (B) Expression levels. The codon adaptation index is an indication of gene expression levels (Sharp and Li, 1987), and the CAI for proteins in each data-set are displayed. The CAI for the P-set is significantly higher than for the As-set (*p*  =  10^−14^, *U*  =  3497.5) and the MS proteome (*p* < 10^−15^, *U*  =  135195.5), whilst the As-set has a higher CAI than the MS proteome (*p* < 10^−15^, *U*  =  146964.5). (C) Molecular weight. The sizes of the proteins in each set are displayed. Proteins in the P-set are significantly smaller than those in the MS proteome (median sizes 28 and 53 kDa respectively; *p*  =  10^−10^, *U*  =  4685) and the As-set (median 58 kDa; *p*  =  10^−9^, *U*  =  11,649). The slight difference in medians between the As-set and the MS proteome is not considered significant. (D) Isoelectric point. The pI values for each data-set are shown. Both the As-set (median pI 6.2) and the P-set (median pI 10.3) are distinct from the MS proteome (median pI 6.8), but in different directions. The As- and P-sets are significantly different from each other (*p*  =  10^−11^, *U*  =  4057.5). The MS proteome has a slightly higher pI value than the As-set (*p*  =  0.001, *U*  =  85,761) and a significantly lower pI value than the P-set (*p*  =  10^−12^, *U*  =  113744.5). (E) Hydrophobicity. The GRAVY scores of each data-set are shown. The As-set has a median GRAVY score of −0.338, which is more hydrophobic than the MS proteome (−0.385; *p*  =  0.006, *U*  =  108,718) but similar to the P-set (−0.334). (F) Secondary structure. Proteins in the As- and P-sets do not show any significant differences in predicted α-helix content, while they have significantly higher α-helix content than the MS proteome (*p*  =  0.0017, *U*  =  134,098 for proteome vs. As-set; *p*  =  0.0011, *U*  =  109,179 for proteome vs. P-set). Proteins in the P-set have a significantly higher β-sheet content than proteins in the As-set (*p*  =  0.0015, *U*  =  10,036) and the MS proteome (*p*  =  4×10^−4^, *U*  =  110,809.5). (G) Intrinsic disorder. A fold-index was calculated where positive values represent proteins likely to be folded, and negative values represent proteins likely to be intrinsically disordered. The As-set (median fold-index 0.12) has a significantly lower proportion of intrinsically disordered proteins than the MS proteome (median fold-index 0.09; *p*  =  0.004, *U*  =  111,594), whereas the P-set has an insignificant increase in disordered proteins. (H) Protein half-lives. The half-lives for proteins in each data-set under non-stress conditions are shown. Proteins in the As- and P-sets have similar half-lives with medians of 67.5 and 84.0 minutes, respectively. These are significantly higher than for proteins in the MS proteome with a median of 53.0 minutes (*p*  =  0.0021, *U*  =  51,996 and *p*  =  0.0012, *U*  =  32805.5, respectively). Stable proteins without a measurable half-life were removed from the analysis. Outliers (> 3rd quartile + 1.5 × IQR or < 1st quartile − 1.5 × IQR) are excluded from all boxplots.

To examine whether these proteins possess particular properties that make them aggregation-prone, we compared them to a list of yeast proteins that are detected by MS in logarithmically growing cells (Washburn et al., 2001). By using these proteins (hereafter called the MS proteome) as background, we avoid potential bias by including proteins that are normally not detected by MS. Aggregated proteins in the P-set are clearly more abundant (*i.e.* present in more molecules/cell), highly expressed (indicated by a high codon adaptation index (CAI)), smaller in size (*i.e.* lower molecular weight (MW)), and have a higher isoelectric point (pI) than proteins in the MS proteome (Fig. 2). These features are consistent with the high proportion of ribosomal proteins in the P-set. Although less pronounced than for the P-set, the As-set is also enriched for abundant and highly expressed proteins (Fig. 2A,B). Arsenite-aggregated proteins have slightly lower pI but do not differ in mean protein size compared to the MS proteome (Fig. 2C,D).

**Fig. 3.**
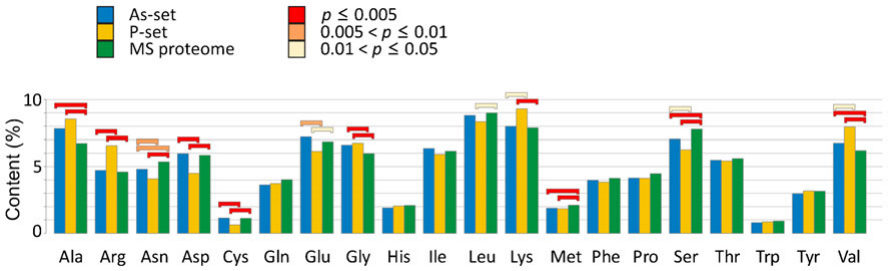
Amino acid composition of aggregation-prone proteins. The relative amino acid composition of aggregated proteins is shown. Relative content is shown for each data-set and the *p*-values for significant differences between the sets are indicated with coloured bars. No bar indicates *p* > 0.05.

The relative amino acid composition of the aggregated proteins differed from that in the MS proteome (Fig. 3); both As- and P-sets are enriched in the aliphatic amino acids glycine, alanine and valine whilst asparagine and serine having polar uncharged side-chains, and methionine are significantly underrepresented. In addition, the P-set is enriched in the basic amino acids lysine and arginine whilst the acidic residues aspartate and glutamate are underrepresented (Fig. 3), in agreement with the higher pI of P-set proteins (Fig. 2D). Consistent with the observed enrichment for aliphatic amino acids (Fig. 3), proteins in the As- and P-sets show a somewhat higher average hydrophobicity (GRAVY score) than those in the MS proteome (Fig. 2E). Proteins in both sets are predicted to have significantly higher α-helix content than the average protein in the MS proteome, and the P-set is additionally enriched for proteins with enhanced β-sheet content (Fig. 2F).

Computational predictions were recently used to group 1822 *S. cerevisiae* proteins (representing ∼27% of the predicted proteome) into four categories; highly structured proteins without aggregation-prone elements (SNA), highly structured proteins with aggregation-prone elements (SA), highly unstructured proteins with non-aggregating lysine/glutamic acid-rich stretches (UNA), and highly unstructured proteins with aggregation-prone glutamine/asparagine-rich stretches (UA) (Gsponer and Babu, 2012). 82 of the aggregation-prone proteins that we identified (representing ∼32% of the proteins in our data-sets) were also present in the Gsponer and Babu data-set. Of those, 69 proteins were predicted to be structured (present in SNA+SA categories) whilst 13 proteins were predicted to be unstructured (present in UNA+UA categories) (supplementary material Table S1). Moreover, the arsenite-aggregated proteins were significantly enriched in the SNA category (supplementary material Table S1) and showed less intrinsic protein disorder than proteins in the P-set and MS proteome (Fig. 2G). The proteins in both P- and As-sets have on the average a longer half-life than proteins in the MS proteome (Fig. 2H), suggesting that these proteins are stable in their folded states, since half-lives were determined by measuring protein abundance over time after inhibition of protein biosynthesis (Belle et al., 2006).

We conclude that high protein expression and abundance, a higher average hydrophobicity, and certain structural properties positively correlate with physiological and arsenite-induced protein aggregation *in vivo*. Moreover, many of these proteins are predicted to be structured and stable in their native folded states.

### Proteins are susceptible for aggregation during translation/folding

**Fig. 4.**
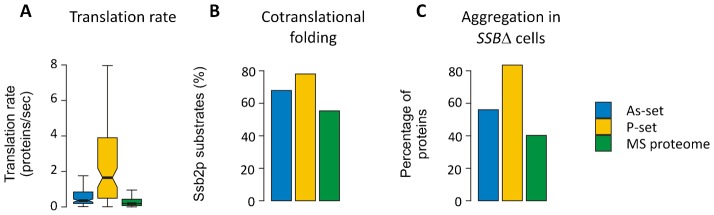
Proteins are susceptible for aggregation during translation/folding. (A) Translation rate. Estimated translation rates per protein species are shown. Proteins in the P-set have a significantly higher translation rate (median 1.6 sec^−1^ per protein species) than proteins in the As-set (0.36 sec^−1^ per species, *p*  =  9×10^−11^, *U*  =  3649) and the MS proteome (0.19 sec^−1^ per species, *p*  =  10^−30^, *U*  =  19,451), while proteins in the As-set have higher translation rates than the MS proteome (*p*  =  10^−13^, *U*  =  49,020). Outliers are not shown. (B) Co-translational folding. Bars indicate the proportion of proteins in the sets that are co-translational substrates of Ssb2p. Both the As-set (68% of proteins, *p*  =  0.005; Fisher's exact test) and the P-set (78%, *p* < 1×10^−6^) have significantly more interactions with Ssb2p than the MS proteome (55%). (C) Aggregation in *SSBΔ* cells. Bars show the proportion of proteins that aggregate in cells lacking Ssb1p and Ssb2p (*SSBΔ*). 56% of the As-set, 83% of the P-set and 40% of the MS proteome aggregate in *SSBΔ* cells and all differences are significant (As-set vs. MS proteome: *p*  =  4×10^−4^, P-set vs. MS proteome: *p* < 10^−15^, As-set vs. P-set: *p*  =  2×10^−6^; Fisher's exact test).

The observations that aggregation-prone proteins are abundant and associated with processes related to translation (Figs 1, 2) prompted us to explore this further. Using data from large-scale translation-rate estimations (Arava et al., 2003), we found that aggregating proteins are translated at a significantly higher rate than proteins in the MS proteome (Fig. 4A). Proteins in the P-set are particularly highly translated with a median translation rate about 8-fold higher than the MS proteome, whilst the As-set proteins have an about 2-fold higher translation rate than those in the MS proteome (Fig. 4A). Many proteins fold during translation, and co-translational folding may be assisted by ribosome-bound chaperones of which Hsp70 is the most prominent. In *S. cerevisiae*, the closely related ribosome-associated Hsp70 proteins Ssb1p and Ssb2p bind cotranslationally to nascent chains and co-translational substrates of Ssb2p were recently identified (Willmund et al., 2013). Both the As-set (68%) and the P-set (78%) are enriched in proteins that are co-translational Ssb2p substrates compared to the MS proteome (55%) (Fig. 4B). This enrichment is even more pronounced when compared to the genome (12% Ssb2p interactors) (supplementary material Fig. S1). Loss of Ssb1p/Ssb2p results in aggregation of newly synthesized proteins (Koplin et al., 2010; Willmund et al., 2013). The overlap between proteins that aggregate in cells lacking Ssb1p/Ssb2p (*SSBΔ*) and those that aggregate in the As- and P-sets is significantly higher than the overlap between proteins that aggregate in *SSBΔ* cells and the proteins in the MS proteome (Fig. 4C). Thus, the majority of the aggregation-prone proteins identified here are substrates of ribosome-associated Hsp70 chaperones. Together with the finding that the proteins in the As- and P-sets are stable in their native state (Fig. 2), these data strongly suggest that proteins are particularly susceptible for aggregation during translation/folding, both during physiological conditions and arsenite exposure.

### Arsenite may inhibit chaperone activity

**Fig. 5.**
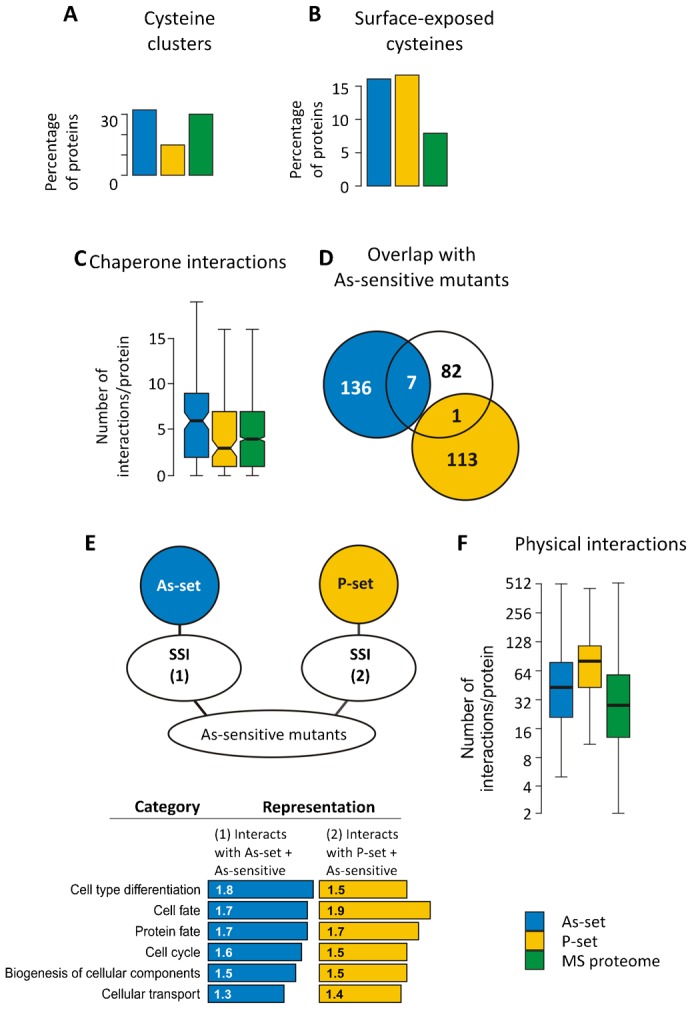
Arsenite toxicity mechanisms. (A) Proportion of proteins containing cysteine clusters (CC, CxC, CxxC or CxxxC). Proteins in the As-set have a similar amount of clusters as the MS proteome, whilst the P-set stands out as significantly lower in cysteine clusters (*p*  =  4×10^−4^, Fisher's exact test). (B) Occurrence of surface-exposed cysteine residues. Proteins in the As-set have a significantly higher proportion of surface-exposed redox reactive cysteines than the genome: 16% of the proteins in the As-set have at least one surface-exposed cysteine in the native fold, compared to 2% of the genome (*p*  =  1.8×10^−13^; binomial test). 13% of the proteins in the P-set have at least one surface-exposed cysteine (*p*  =  4.5×10^−8^). Proteins in the As-set and P-set do not differ significantly from each other (*p*  =  0.5, *G*  =  0.40; G-test of independence without continuity correction). (C) Number of chaperone interactions per protein. All differences are significant: As-set vs. P-set, *p*  =  0.05; As-set vs. MS proteome, *p*  =  10^−20^; P-set vs. MS proteome, *p*  =  3×10^−6^; Student's t-test. Error bars represent S.D. (D) Overlap between aggregated proteins and arsenite-sensitive mutants. 90 deletion mutants were shown to be arsenite-sensitive in three independent studies (Thorsen et al., 2009). These protective proteins are not significantly overrepresented in the As-set or P-set (*p*  =  0.8, 10^6^ permutations). (E) Synthetic sick interactions (SSI) between As- or P-set and As-sensitive deletion mutants. Genes that have SSI with the As-set and with As-sensitive deletion mutants, as well as genes that have SSI with the P-set and with As-sensitive deletion mutants were extracted. Most significant functional enrichments compared to genome content are shown (genome  =  1). (F) Physical interactions. Proteins in the As-set have on average 43 physical interactions, compared to proteins in the MS proteome that have 28 physical interactions (*p*  =  0.00034). Proteins in the P-set have on average 80.5 physical interactions (*p* < 10^−6^).

We next sought to gain insights into arsenite-induced protein aggregation by identifying features that distinguish the As-set from the P-set. Arsenite has high reactivity with sulphydryl groups and readily forms metal–thiol bonds with vicinal cysteines in proteins (Delnomdedieu et al., 1993). We reasoned that cysteine residues may be exposed in nascent polypeptides and targeted by arsenite for aggregation before folding is accomplished. To test this prediction, we explored whether the As-set is enriched for cysteine-rich proteins. Unexpectedly, the relative amount of cysteine was similar in the As-set and the MS proteome, whereas cysteine was underrepresented in the P-set (Fig. 3). Moreover, 8% of the proteins in the As-set and 9% in the MS proteome lack cysteines, while 31% of proteins in the P-set have no cysteines. Likewise, the As-set was not enriched for proteins with vicinal cysteines; 32% of the arsenite-aggregated proteins contained at least one CC, CxC, CxxC or CxxxC motif, compared to 30% in the MS proteome and 15% in the P-set, the latter being significantly lower than the MS proteome (Fig. 5A). Proteins having surface-exposed cysteines in their native fold were overrepresented to a similar extent in both the As-set and P-set (Fig. 5B), indicating that proteins with surface-exposed cysteines are generally susceptible for aggregation. Hence, arsenite-induced protein aggregation cannot be explained by a simple model where this metalloid targets cysteine-rich nascent proteins for aggregation.

Our previous data indicated that arsenite affects chaperone-mediated protein folding *in vivo* (Jacobson et al., 2012), and our current study suggested that a large fraction of arsenite-aggregated proteins are co-translational substrates of Ssb2p (Fig. 4B). To further explore the impact of arsenite on chaperones, we scored the number of interactions between proteins in our data-sets and the 63 chaperones present in *S. cerevisiae* (Gong et al., 2009). Interestingly, proteins in the As-set are engaged in significantly more chaperone interactions per protein than the MS proteome, the protein-coding genome or the proteins in the P-set (Fig. 5C). Moreover, a larger fraction of arsenite-aggregated proteins (88%) interact with chaperones than the proteins in the P-set (77%), the MS proteome (77%) or the protein-coding genome (57%) (supplementary material Fig. S2). The finding that arsenite-aggregated proteins are enriched for multiple chaperone interactions, supports the notion that chaperone inhibition by this metalloid can lead to extensive protein aggregation *in vivo* (Jacobson et al., 2012).

### Protein aggregation and arsenite toxicity

We recently showed that arsenite-induced protein aggregation is correlated with toxicity of this metalloid (Jacobson et al., 2012), but how these aggregates affected cell viability was not fully elucidated. Here, we examined three mutually non-exclusive models of how aggregation may contribute to arsenite toxicity: (1) by inactivating/depleting individual proteins with protective or detoxification functions, (2) by inactivating/depleting proteins acting in parallel pathways thereby producing a more severe phenotype than expected from inactivation of the corresponding individual proteins (*i.e.* by synergistic or synthetic effects), and/or (3) by a seeding effect caused by aberrant interactions of the aggregated protein with many other proteins. The overlap between arsenite-aggregated proteins and a set of gene deletions that cause arsenite sensitivity (Thorsen et al., 2009) was poor (*p*  =  0.8; 10^6^ permutations; Fig. 5D), suggesting that inactivation/depletion of individual protective proteins may not be a major toxicity mechanism. Accordingly, the As- and P-sets are not substantially different from the MS proteome regarding the proportion of essential proteins (supplementary material Table S2).

To assess the importance of synthetic effects, we evaluated the number of genetic interactions for each protein in the data-sets. The total number of genetic interactions between the As-set or the P-set and the genome was not significantly different from the number of genetic interactions between the MS proteome and the genome (supplementary material Fig. S3A). Likewise, the number of negative genetic or synthetic sick interactions (SSI) between the As-set and the genome was not significantly different from that between the MS proteome and the genome (supplementary material Fig. S3B,C). In contrast, the P-set was enriched for SSI with the genome (median of 8 SSI/protein) compared to that between the MS proteome and the genome (median 3 SSI/protein) (supplementary material Fig. S3B). We next analysed whether deletions that cause arsenite sensitivity could be candidate genes for negative genetic interactions with arsenite-aggregating proteins. However, both As- and P-sets had fewer SSI with genes whose deletion causes arsenite-sensitivity compared to the number of SSI between the MS proteome and the arsenite-sensitive gene deletion mutants (supplementary material Fig. S3B). The genes that show SSI with the As- or P-sets and the arsenite-sensitive gene deletion mutants are enriched for similar functions including protein fate (folding, modification, destination), cell cycle, cellular differentiation and cell fate (Fig. 5E). Taken together, synthetic effects do not appear to be a major contributor to arsenite toxicity.

We next scored the number of physical interactions for each protein in the data-sets. Interestingly, members of both the As- and P-sets are engaged in a significantly higher number of protein–protein interactions (PPI) per protein than the proteins in the MS proteome. Comparison of the number of PPI between the As-set and the genome with that between the MS proteome and the genome revealed a strong overrepresentation for the As-set (median of 43 PPI/protein for the As-set *vs.* 28 PPI/protein for the MS proteome). The overrepresentation for the P-set is even more extreme (median of 80.5 PPI/protein) (Fig. 5F). The observation that aggregation-prone proteins are enriched for multiple PPI supports model 3; misfolded forms of these proteins might engage in extensive aberrant protein–protein interactions during arsenite exposure, thereby committing other proteins to misfold and aggregate and affecting cell viability.

### Expression of aggregation-prone proteins is decreased during arsenite stress

**Fig. 6.**
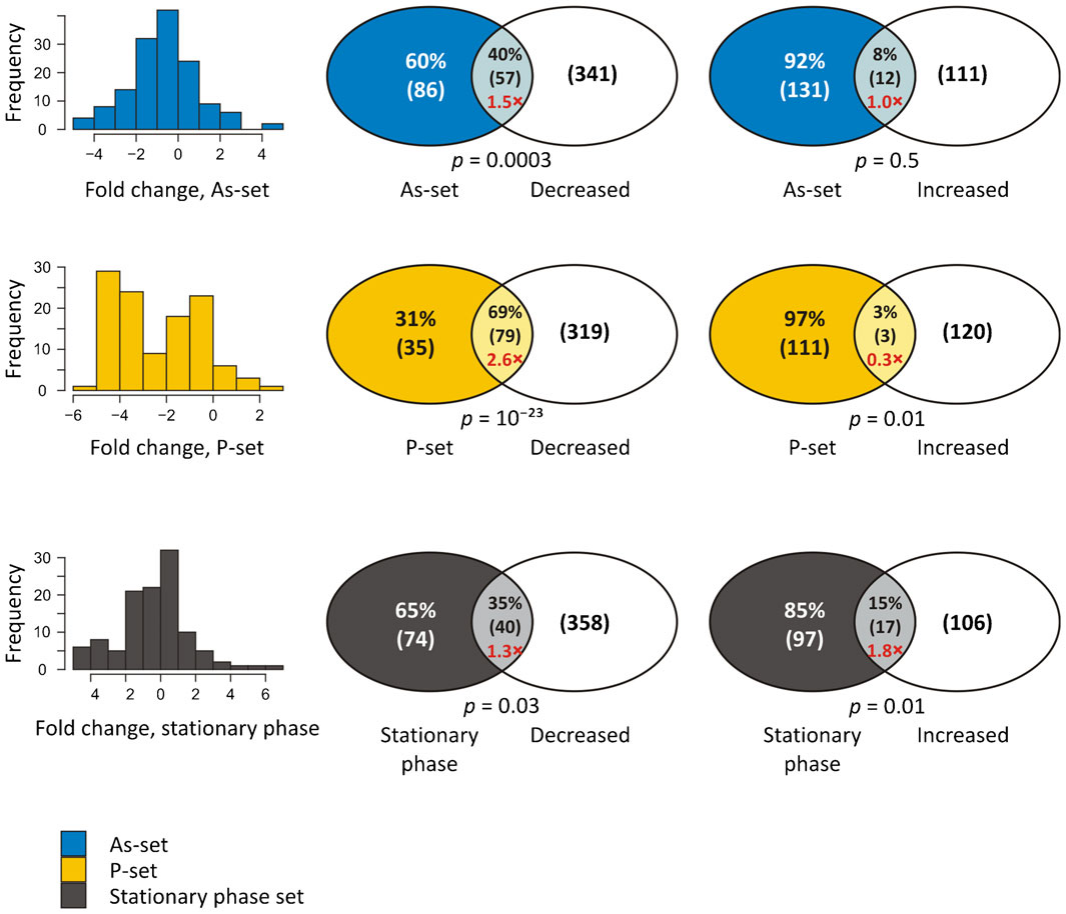
Expression of aggregation-prone proteins is decreased during arsenite exposure. The histograms show the relative change in expression for proteins in the As-set, P-set and stationary phase aggregates in response to 1.0 mM arsenite (Thorsen et al., 2007). The As-set and P-set are notably shifted toward decreased expression. The Venn diagrams show the overlap between the data-sets and proteins with at least a 2-fold change in gene expression following arsenite exposure. Relative numbers give the proportion of proteins that are differentially expressed or that show a less than 2-fold change in gene expression. Numbers in parentheses give the absolute number of proteins in the intersection and the representation factor, *i.e.* observed/expected, is shown in red. *p*-values were calculated with hypergeometric tests using the MS proteome as background. The As-set is shifted toward lower expression, having 50% more proteins with ≥ 2-fold lower expression than expected (*p*  =  0.0003, hypergeometric test). Proteins in the P-set show a decreased expression with 69% having ≥ 2-fold decreased expression, which is 2.6 times more than expected as compared to the MS proteome (*p*  =  10^−23^), and 3% having a ≥ 2-fold increased expression, which is only one third of what is expected. Proteins that aggregate in stationary phase show no consistent trend, with proteins being both up- and down-regulated during arsenite exposure.

Given that misfolded/aggregated proteins may be cytotoxic, we asked whether cells regulate expression of aggregation-prone proteins during conditions that cause widespread misfolding and aggregation. For this, we compared the set of proteins that aggregated in response to arsenite (the As-set) to a set of proteins that show differential gene expression during arsenite exposure (Thorsen et al., 2007). In response to arsenite, 1080 genes showed a >2-fold differential (induced and decreased) expression, corresponding to 17% of the genome (Fig. 6). The equivalent number for the MS proteome is 521 genes, corresponding to 27% of the proteins with ≥ 2-fold decreased expression and 8% with ≥ 2-fold increased expression. Compared to the MS proteome, proteins in the As-set showed a significantly decreased gene expression (40% with ≥ 2-fold decrease) whereas there was no correlation between gene induction and protein aggregation. Decreased gene expression following arsenite exposure was even more pronounced for the P-set proteins (69% with ≥ 2-fold decrease). This down-regulation would make sense since the P-set proteins also aggregate during arsenite exposure. We expanded the analysis by including a set of 114 yeast proteins that aggregated in stationary phase and reverted to a soluble form upon nutrient re-addition (Narayanaswamy et al., 2009). These reversible assemblies appear to represent storage depots of functional proteins (Narayanaswamy et al., 2009; Petrovska et al., 2014). The stationary phase set had 22 proteins in common with the As-set and 16 in common with the P-set (supplementary material Fig. S4). In the stationary phase set, there was no strong correlation between gene induction or repression and protein aggregation during arsenite exposure (Fig. 6). Taken together, expression of aggregation-prone proteins is decreased following arsenite exposure. Moreover, cells may regulate gene expression differently during acute proteotoxic stress caused by arsenite and during a slow(er) progression into stationary phase.

### Human orthologues of aggregated proteins

Protein misfolding and aggregation is associated with several neurodegenerative and age-related disorders including Alzheimer's disease (AD), amyotrophic lateral sclerosis (ALS) and Parkinson's disease (PD). In these diseases, specific proteins adopt non-native conformations and aggregate. In addition, aberrant interactions between disease-associated and other cellular proteins might lead to extensive co-aggregation and loss of function of non-disease proteins (Hartl et al., 2011; Stefani and Dobson, 2003). Several of the aggregation-prone yeast proteins identified in this current study have human or mouse orthologues that are implicated in protein folding disorders and/or co-aggregate with specific folding disease-associated proteins in AD (Liao et al., 2004; Wang et al., 2005), familial ALS (Basso et al., 2009) or PD (Xia et al., 2008) (supplementary material Table S4). Interestingly, yeast orthologues of disease-associated proteins are overrepresented among the aggregated proteins identified in this study. Considering that the As-set and P-set together constitute 3.9% of the total yeast genome, it is noteworthy that this set contains 50% more orthologues to β amyloid-associated aggregates (AD) than the genome (*p*  =  2×10^−9^), whereas the corresponding number is 34% for orthologues to proteins that are present in human neurofibrillary tangles (AD) (*p* < 1×10^−15^). Likewise, the set of aggregated yeast proteins contains 13% more orthologues to proteins that co-aggregate with α-synuclein in PD (*p* < 1×10^−15^) than the genome, and 19% more orthologues to aggregating proteins in a familial ALS mouse model (*p* < 1×10^−15^) than the genome. These findings suggest that the basic mechanisms that govern protein aggregation in yeast may be relevant also during human disease processes.

## DISCUSSION

In this study, we addressed fundamental questions related to protein aggregation under physiological conditions and arsenite exposure. Our analyses suggest that highly expressed proteins are particularly susceptible for aggregation and that cells invest significant resources to ensure their solubility. Our results also suggest that arsenite specifically interferes with cotranslational protein folding and that arsenite-aggregated proteins engage in many protein–protein interactions which may contribute to the toxicity of this metalloid.

### Characteristics of the aggregation-prone yeast proteome

The yeast proteins identified in this current study are abundant, have extensive physical interactions, and possess certain structural properties that may increase their susceptibility for aggregation *in vivo*. Some of these properties, such as high hydrophobicity and β-sheet content, were previously associated with protein aggregation (Hartl et al., 2011; Stefani and Dobson, 2003). For example, aliphatic amino acids like glycine, alanine and valine were overrepresented in our data-sets (Fig. 3) as well as in sequences with high aggregation propensity, in sequences that promote fibril formation of disease-aggregating proteins, and in proteins that aggregate in *C. elegans* during ageing (David et al., 2010; Du et al., 2003; Goldschmidt et al., 2010; Lansbury et al., 1995; Teng and Eisenberg, 2009). Consistent with this enrichment, the proteins in the As- and P-sets were somewhat more hydrophobic than those in the MS proteome (Fig. 2E). Unlike yeast prion proteins and human Huntingtin, the aggregated proteins in our data-sets were neither rich in glutamine and asparagine (Fig. 3), nor did they have expanded glutamine repeats (data not shown). Our data-sets were enriched for proteins with high α-helix (As- and P-sets) and β-sheet (P-set) content (Fig. 2F). Likewise, proteins that aggregate during ageing in *C. elegans* have a high propensity to form β-sheets (David et al., 2010) and numerous disease-related aggregates contain β-rich amyloid structures (Stefani and Dobson, 2003). It remains to be determined whether the aggregates identified here are structured or amorphous.

Highly expressed proteins are predicted to be more soluble and less aggregation-prone than other proteins, based on the finding that *in vivo* expression levels of human genes are anti-correlated with the *in vitro* aggregation rates of the corresponding proteins (Tartaglia et al., 2007). Here, we found a correlation between high protein abundance and high aggregation propensity *in vivo*. Assuming a constant error rate during translation/folding (Drummond and Wilke, 2009), highly expressed and abundant proteins are more likely to encounter errors per protein species resulting in misfolding and aggregation than weakly expressed proteins. At the same time, aggregating proteins in both As- and P-sets were enriched for multiple chaperone interactions (Fig. 5C), indicating that high expression is counterbalanced by molecular chaperones to allow soluble expression. A large fraction of the aggregated proteins identified here are lysine- and arginine-rich ribosomal proteins (Figs 1, 3) that are known to easily aggregate if the highly basic patches are not appropriately shielded by chaperones (Jäkel et al., 2002). Indeed, the general chaperone network, as well as specific factors, protects ribosomal proteins from aggregation during synthesis, nuclear import and ribosome assembly (Albanèse et al., 2010; Jäkel et al., 2002; Koch et al., 2012; Koplin et al., 2010).

We provide evidence that proteins are susceptible for aggregation primarily during translation/folding: (1) functions related to protein biosynthesis and translation were enriched among aggregated proteins, (2) high translation rates were associated with increased aggregation propensity, and (3) a large proportion of the aggregated proteins are co-translational substrates of ribosome-associated Hsp70 Ssb2p and aggregate in the absence of Ssb1p/Ssb2p. Consistently, loss of ribosome-associated chaperones (yeast) or the chaperonine GroEL (*E. coli*) has been shown to cause extensive aggregation of nascent proteins (Chapman et al., 2006; Koplin et al., 2010; Willmund et al., 2013). Folding of nascent chains cannot be completed until all protein domains have been synthesized (Hartl et al., 2011; Stefani and Dobson, 2003). Our data-sets were enriched for proteins with high α-helix and β-sheet content (Fig. 2F), suggesting that these multi-domain proteins may need longer time to reach their native fold and supports the notion that proteins are particularly susceptible for aggregation while being translated or folded *in vivo*. Biophysical studies indicated that folded proteins need to (partially) unfold and expose aggregation-prone sequences to facilitate aggregation (Stefani and Dobson, 2003). Specific *in vivo* conditions may induce extensive unfolding and aggregation of native proteins, such as high temperature. The proteins in our data-sets appear relatively stable in their native (folded) state (Fig. 2; supplementary material Table S1). Thus, large-scale protein unfolding as a general cause of aggregation *in vivo* appears unlikely, at least under physiological growth and arsenite exposure. Taken together, our analyses indicate that in living cells, newly translated proteins presumably in a non-native form that exposes aggregation-prone sequences, are at a high risk of aggregation before they reach a stable native conformation.

### Protein aggregation and toxicity during arsenite stress

Proteins in the As- and P-sets have several characteristics in common. However, the features that distinguish the As-set from the MS proteome were often less pronounced than for the P-set (*e.g.* protein expression and abundance, translation rate, secondary structure; Figs 1–4) and an extended set of proteins aggregated following arsenite exposure (supplementary material Table S3). These data suggest that arsenite may lower the overall ‘threshold’ for protein aggregation and that the inclination of a given protein to aggregate increases during exposure. Unexpectedly, arsenite-aggregated proteins were not enriched for cysteine-rich proteins or for proteins with vicinal cysteine pairs (Figs 3, 5). Hence, our analysis does not support a simple model in which arsenite targets exposed cysteine residues in nascent cysteine-rich polypeptides. Nevertheless, given that aggregation-prone proteins are abundant, we cannot exclude that this mechanism contributes to the toxic action by this metalloid. Importantly, the As- and P-sets are enriched for multiple chaperone interactions (Fig. 5C), indicating a high demand of chaperone assistance for proper folding of these proteins. Together with our previous findings (Jacobson et al., 2012), these data are consistent with a model in which arsenite causes widespread protein aggregation by interfering with chaperone activity. The As- and P-sets were enriched for proteins with surface-exposed cysteines (Fig. 5B). Interestingly, the ribosome-associated Ssb1p and Ssb2p as well as the cytosolic Ssa1p chaperones contain surface-exposed cysteines (Marino et al., 2010) and were present in the arsenite-aggregated protein fraction (Jacobson et al., 2012) (supplementary material Table S3). Thus, arsenite might target these chaperones for inactivation and/or aggregation, thereby diminishing the overall folding capacity of the cell and eliciting accumulation of misfolded and aggregated proteins. It will be important to identify arsenite-targeted chaperones to fully understand how this metalloid causes aggregation.

How does protein aggregation contribute to arsenite toxicity? There was no correlation between aggregation of a given protein and arsenite-sensitivity of the corresponding gene deletion mutant (Fig. 5D). Likewise, synthetic interactions do not appear to be a major contributor to arsenite toxicity (Fig. 5E; supplementary material Fig. S3). Instead, proteins in the As- and P-sets were enriched for multiple protein–protein interactions (Fig. 5F). Hence, misfolded/aggregated forms of these proteins might engage in extensive aberrant protein–protein interactions during arsenite exposure thereby affecting cell viability. Such aberrant interactions could be numerous given that aggregation-prone proteins are highly expressed and translated at high rates in cells. This model is in agreement with our previous *in vitro* data showing that arsenite-aggregated proteins can act as seeds, committing other proteins to misfold and aggregate (Jacobson et al., 2012). Alternatively, arsenite may interfere with chaperones specifically because it selectively affects proteins with high chaperone demands. In this model, arsenite-induced aggregates would be toxic because they cause a rapid depletion of chaperone pools.

### Regulation of aggregation-prone proteins during stress conditions

We show that expression of the majority of the aggregation-prone proteins in the As- and P-sets is decreased in response to arsenite exposure (Fig. 6). This down-regulation could be a result of inhibition of global protein synthesis by arsenite (Brostrom and Brostrom, 1997; Liu et al., 2013; Simpson and Ashe, 2012), since the As- and P-sets are enriched for highly expressed genes. However, how cells sense and signal disturbed protein homeostasis to the translational and transcriptional machineries to avoid excessive aggregation is poorly understood. Yeast cells respond to many stress conditions, including arsenite, by strongly decreasing expression of ribosomal protein-encoding genes (Gasch et al., 2000; Thorsen et al., 2007). This response is vital as a large part of the cellular resources are devoted to ribosomal protein synthesis (Warner, 1999). In addition to save resources, our results suggest that this response may be important to avoid excessive protein aggregation during arsenite exposure. The following observations support this notion: (1) inhibiting translation with cycloheximide prevents formation of aggregates during arsenite exposure and improves arsenite tolerance (Jacobson et al., 2012), (2) many aggregation-prone proteins are ribosomal proteins (Fig. 1) and expression of ribosomal genes is down-regulated at arsenite concentrations that induce protein aggregation but does not affect growth to any large extent (Jacobson et al., 2012; Thorsen et al., 2007). It is possible that other misfolding-promoting conditions elicit a similar response. Interestingly, our data also suggest that cells regulate gene expression differently during acute proteotoxic stress caused by arsenite and during a slow(er) progression into stationary phase (Fig. 6). The cellular sensing and signalling mechanisms that control these responses remain to be understood.

### In vivo aggregation vs. computational predictions

Computational predictions suggested that yeast proteins with high intrinsic potential to aggregate have low synthesis, low abundance and high turnover compared to non-aggregating proteins (Gsponer and Babu, 2012). This is in contrast to the properties associated with *in vivo* protein aggregation presented here; aggregation-prone proteins were abundant, highly translated and have a longer half-life than the MS proteome. Thus, computational tools that are based on a limited set of rules cannot capture the complex and crowded intracellular environment in which proteins need to fold and assemble in order to carry out their biological functions, often in interaction with other proteins, various macromolecules or metabolites (Vendruscolo, 2012). Our current study suggests that high protein abundance and failure rates during translation/folding are critical factors that contribute to protein aggregation in living systems. Moreover, our analyses indicate that high expression is counterbalanced by molecular chaperones to allow soluble protein expression. These factors act in addition to well-described intrinsic aggregation parameters and distinguish aggregation-prone proteins from the average proteome. While preparing this manuscript for submission, Vendruscolo and co-workers proposed that abundant proteins are at a higher risk of aggregation and that their solubility must be maintained by the PQC system. These ‘supersaturated’ proteins represent a substantial fraction of the proteome and are overrepresented in processes associated with neurodegenerative disorders (Ciryam et al., 2013). Our analyses support these predictions; abundant proteins are at high risk to aggregate, are enriched for multiple chaperone interactions, and are stable in their native, folded states.

### Conclusions

This study provided novel and extended insights into the rules that govern protein aggregation in living cells and a framework to elucidate the underlying mechanisms. Protein aggregation is a molecular hallmark of a number of pathological conditions including neurodegenerative and age-related disorders. Remarkably, we found several homologues of aggregation-prone yeast proteins to be present in human disease-associated aggregates in AD, ALS, and PD (supplementary material Table S4). Likewise, an overlap between ageing-dependent aggregation in *C. elegans* and disease-dependent aggregation in mammals has been reported (David et al., 2010). Finally, protein abundance and solubility underlies physiological and arsenite-induced protein aggregation in living yeast cells and is associated with neurodegenerative disorders (Ciryam et al., 2013). Thus, the underlying mechanisms of protein aggregation appear to be evolutionarily conserved and similar rules may apply in disease and non-disease settings.

## Supplementary Material

Supplementary Material
