# Supplementary material for: Global analysis of protein aggregation in yeast during physiological conditions and arsenite stress
Source: Biol Open. 2014 Sep 12;3(10):913–23. doi: 10.1242/bio.20148938 (PMC4197440; doi:10.1242/bio.20148938)
Supplement: Supplementary Material [file supp_3_10_913__index.html]

Global analysis of protein aggregation in yeast during physiological conditions and arsenite stress — Supplementary Material 

# Global analysis of protein aggregation in yeast during physiological conditions and arsenite stress

## bio.20148938 Supplementary Material

**Files in this Data Supplement:**

- Supplementary Material - Sebastian Ibstedt et al. doi: 10.1242/bio.20148938
- Table S3 - List of aggregated proteins and aggregation-associated parameters
- Table S4 - List of yeast proteins and their human or mouse orthologues that aggregate in disease processes
