# Supplementary material for: Global analysis of protein aggregation in yeast during physiological conditions and arsenite stress
Source: Biol Open. 2014 Sep 12;3(10):913–23. doi: 10.1242/bio.20148938 (PMC4197440; doi:10.1242/bio.20148938)
Supplement: Supplementary Material [file supp_bio.20148938_bio.20148938-s1.pdf]

Biology Open (2014) 000, 1–11 doi:10.1242/bio.20148938

Supplementary Material

Sebastian Ibstedt et al. doi: 10.1242/bio.20148938

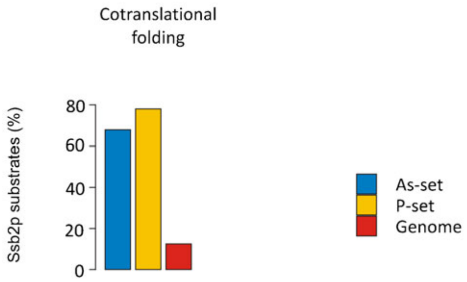

Fig. S1. Co-translational substrates of ribosome-associated Hsp70 (Ssb2p). Bars indicate the proportion of aggregated proteins that are co-translational substrates of Ssb2p. Both the As-set (68% of proteins, $p < 1{\times}10^{-15}$) and the P-set (78%, $p < 1{\times}10^{-15}$) have more interactions with Ssb2p than the genome (12%).

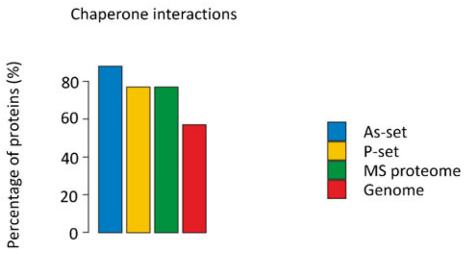

Fig. S2. Chaperone interactions. Histograms show the proportions of proteins in the data-sets that interact with chaperones. 88% of the proteins in the As-set interact with at least one chaperone. This is more than the P-set (77%; $p = 0.028$), the MS proteome (77%; $p = 0.0015$) and the genome (57%; $p = 1{\times}10^{-15}$). Comparisons between groups were made with Fisher's exact test.

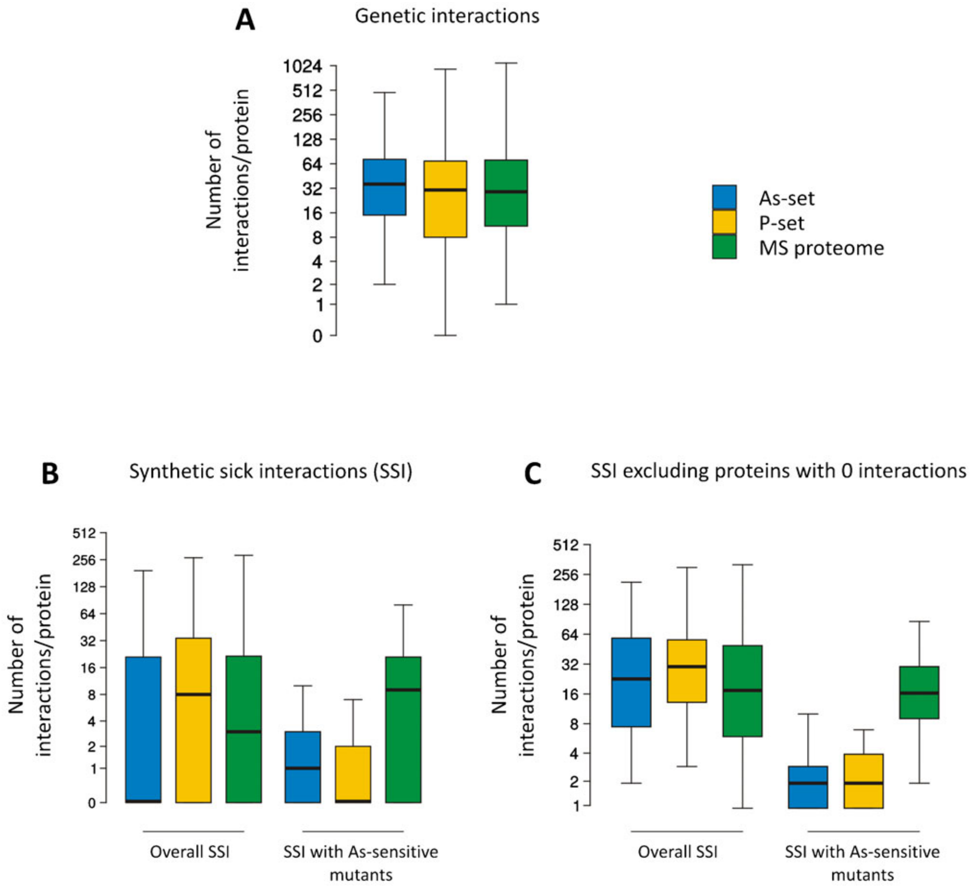

Fig. S3. Genetic interactions. (A) Genetic interactions for the As-set, P-set and MS proteome. No significant difference in genetic interactions per protein is seen between the data-sets. (B) Synthetic sick interactions (SSI) for the As-set, P-set and MS proteome. Synthetic sickness was defined as $|f_{ab} - f_a \times f_b| > 0.088$, where f_{ab} is the fitness of the double mutant and f_a and f_b is the fitness of the single mutants. The first three boxplots show total SSI per gene, while the last three boxplots show the number of SSI between genes in each data-set and genes whose deletion result in arsenite-induced fitness defects. The P-set has a significantly higher median amount of total SSI (8 per gene) than the MS proteome (3 per gene; $p = 0.012$), but genes in both the As-set and P-set have fewer SSI with genes that contribute to As-tolerance than genes in the MS proteome have. (C) SSI as in panel B) but excluding proteins that lack interactions. The As-set and P-set still have fewer SSI with genes that contribute to As-tolerance than genes in the MS proteome have.

Biology Open (2014) 000, 1–11 doi:10.1242/bio.20148938

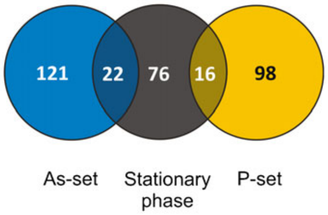

Fig. S4. Overlap between protein aggregates formed during physiological conditions, arsenite exposure and in stationary phase. Stationary phase aggregates are largely distinct from aggregates formed during physiological conditions and arsenite exposure.

Table S1. Aggregation-prone proteins are predicted to be structurally stable

	SNA	SA	UNA	UA	tot
**Absolute**					
As	35	8	11	1	143
P	20	6	1	0	114
MS	241	103	83	57	1475
tot	716	711	198	197	
**Relative**					
As	24.5%	5.6%	7.7%	0.7%	
P	17.5%	5.3%	0.9%	0.0%	
MS	16.3%	7.0%	5.6%	3.9%	

The majority of the aggregation-prone proteins that we identified are predicted to be structured according to the classification by (Gsponer and Babu, 2012) and the arsenite-aggregated proteins (As-set) are significantly enriched in the SNA category. SNA: highly structured proteins without non-aggregation prone elements; SA: highly structured proteins with hydrophobic domains that are likely to form β-sheet aggregates; UNA: unstructured proteins with K/E-rich stretches that decrease aggregation propensity; UA: unstructured aggregation-prone proteins with Q/N-rich stretches.

Table S2. Essential proteins

	As-set	P-set	MS-proteome
Essential	51	35	375
Non-essential	92	79	990
Total	143	114	1365
Essential (%)	35.7	30.7	27.5

The As-set contains relatively more essential proteins than the MS proteome ($p = 0.04$, Fisher's exact test) whereas the P-set is not different from the MS proteome.
